# Blood–brain barrier disruption in CCL2 transgenic mice during pertussis toxin-induced brain inflammation

**DOI:** 10.1186/2045-8118-9-10

**Published:** 2012-04-30

**Authors:** Angela E Schellenberg, Richard Buist, Marc R Del Bigio, Henrik Toft-Hansen, Reza Khorooshi, Trevor Owens, James Peeling

**Affiliations:** 1 Department of Pharmacology & Therapeutics, University of Manitoba, 753 McDermot Avenue, Winnipeg, Manitoba, R3E 0T6, Canada; 2 Department of Radiology, University of Manitoba, 753 McDermot Avenue, Winnipeg, Manitoba, R3E 0T6, Canada; 3 Department of Pathology, University of Manitoba, 715 McDermot Avenue, Winnipeg, Manitoba, R3E 3P4, Canada; 4 Department of Clinical Immunology, Odense University Hospital, University of Southern Denmark, Sdr. Boulevard 29, 5000, Odense C, Denmark; 5 Hans Christian Andersen Children’s Hospital, Odense University Hospital, Sdr. Boulevard 29, 5000, Odense C, Denmark; 6 Department of Neurobiology Research, Institute of Molecular Medicine, University of Southern Denmark, J. B. Winsløwsvej 25, 2. Sal, 5000, Odense C, Denmark; 7 Neuroimmunology Unit, Montreal Neurological Institute, McGill University, Montréal, Québec, Canada

**Keywords:** Chemokine, Central nervous system, Inflammation, Multiple sclerosis, Magnetic resonance imaging

## Abstract

**Background:**

The chemokine CCL2 has an important role in the recruitment of inflammatory cells into the central nervous system (CNS). A transgenic mouse model that overexpresses CCL2 in the CNS shows an accumulation of leukocytes within the perivascular space surrounding vessels, and which infiltrate into the brain parenchyma following the administration of pertussis toxin (PTx).

**Methods:**

This study used contrast-enhanced magnetic resonance imaging (MRI) to quantify the extent of blood–brain barrier (BBB) disruption in this model pre- and post-PTx administration compared to wild-type mice. Contrast-enhanced MR images were obtained before and 1, 3, and 5 days after PTx injection in each animal. After the final imaging session fluorescent dextran tracers were administered intravenously to each mouse and brains were examined histologically for cellular infiltrates, BBB leakage and tight junction protein.

**Results:**

BBB breakdown, defined as a disruption of both the endothelium and glia limitans, was found only in CCL2 transgenic mice following PTx administration and seen on MR images as focal areas of contrast enhancement and histologically as dextrans leaking from blood vessels. No evidence of disruption in endothelial tight junctions was observed.

**Conclusion:**

Genetic and environmental stimuli were needed to disrupt the integrity of the BBB in this model of neuroinflammation.

## Background

Multiple sclerosis (MS) is an autoimmune disease of the central nervous system (CNS), wherein inflammation, demyelination, and blood–brain barrier (BBB) disruption occurs. The magnetic resonance imaging (MRI) contrast agent gadolinium diethylenetriaminepentaacetate (Gd-DTPA) does not move through the intact BBB. Following intravenous administration of Gd-DTPA, a signal increase on T_1_-weighted MR images is observed where the BBB is disrupted at sites of inflammation [1,2]. Histological studies using tracers [3-5] and contrast-enhanced MRI [6-9] have both shown that opening of the BBB is an early event in the development of the disease, though the exact mechanism that causes BBB disruption is currently unknown.

The chemokine CCL2, previously known as monocyte chemoattractant protein-1, has a role in the recruitment of inflammatory cells into the CNS [10,11]. CCL2 is expressed within active and chronic MS lesions [11-14]. Astrocytes [13-15] and microglia [16] are believed to be sources of CCL2. Leukocytes accumulate in the perivascular space between the endothelium and glia limitans (formed by astrocytic foot processes) in transgenic mice that overexpress CCL2 in the CNS [17,18]. This transgenic mouse model is a unique experimental system for studying the infiltration of inflammatory cells across the endothelium and glia limitans of the BBB, which is not possible in EAE [19]. These mice show infiltration of leukocytes across the BBB following pertussis toxin (PTx) administration along with an upregulation of proinflammatory cytokines and metalloproteinase genes [18]. This metalloproteinase-dependent migration of inflammatory cells across the glia limitans supports the hypothesis that BBB leakage is present in this model. PTx increases the permeability of the BBB in animal models of EAE and results in a more severe disease course [20].

The integrity of the BBB in mice overexpressing CCL2 in the CNS pre- and post-PTx compared to wild-type mice is currently unknown. Information regarding BBB permeability to small and large molecules in this model of inflammation may provide insights into the individual and combined effects of CCL2 upregulation and PTx administration, and thus into the overall mechanism of neuroinflammation. T_1_-weighted contrast-enhanced MRI was used to determine how treatment with PTx affects BBB permeability in PTx and vehicle treated CCL2 transgenic and wild-type mice. This study seeks to determine the permeability of a small molecular sized MR contrast agent (550D) and large dextran tracer (70kD) across the BBB in the CCL2 transgenic mouse model of inflammation and considers the potential implications of these findings in encephalomyelitis.

## Methods

### Animal model

Female and male MBP-JE transgenic mice 11–13 weeks old (bred at the University of Southern Denmark) overexpressing CCL2 in the CNS under control of a myelin basic protein promoter, [17,18] were given a single intraperitoneal injection of PTx (Cedarlane Laboratories, Hornby, ON) at a dose of 10 μg/kg (n = 9) or 20 μg/kg (n = 7). PTx stock solution (50 μg/mL in phosphate-buffered saline) was diluted to 1 μg/mL or 2 μg/mL using Hanks’ balanced salt solution (HBSS; Invitrogen Corporation, Burlington, ON) for doses of 10 μg/kg or 20 μg/kg respectively. Transgenic mice that received the equivalent volume of HBSS (n = 6) served as controls. Similarly, a group of female and male wild-type B6D2F1 mice 11 weeks old (Charles River Canada, Saint Constant, QC) were given an injection of either 10 μg/kg PTx (n = 6), 20 μg/kg PTx (n = 5), or HBSS (n = 5) as. The B6D2F1 mice are the most appropriate control strain for this experiment, since the transgenic mice were generated by injecting transgenes into the eggs from a cross between C57BL/6 J × DBA/2 mice as previously described [17] and are maintained by brother-sister mating. All mice were housed in pathogen-free conditions with free access to food and water, and were weighed and monitored daily. All animal procedures were approved by the Animal Care Committee at the University of Manitoba.

### MR imaging

**Figure 1:**
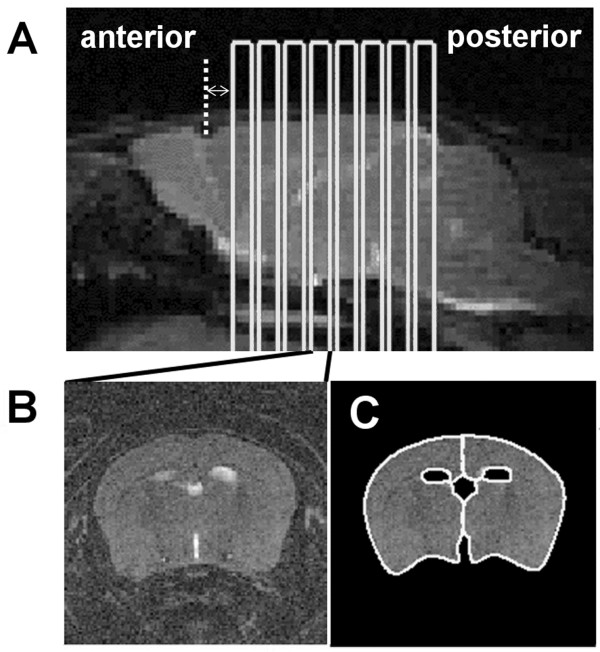
(**A**) Sagittal MR image of a mouse brain showing the slice positioning for MR imaging. The slice furthest anterior was positioned 1 mm posterior to the notch at the olfactory bulbs indicated by the dotted line. (**B**) Coronal T_2_-weighted MR images (TE = 80.4 ms) were used to define the area of the brain omitting the enhancing ventricles seen in (**C**).

T_2_-weighted MR images and T_1_-weighted contrast-enhanced MR images of the brain were obtained using a Bruker Biospec imager with a 7 T/21 cm magnet and a quadrature volume coil 2 cm in diameter. Mice were anesthetized by ventilating with 5 % isoflurane in 2:3 O_2_/N_2_O for induction and maintained using 1.5-2.0 % isoflurane delivered via a nose cone during imaging. Eight T_2_-weighted MR images spanning the brain were obtained using a multi-slice multi-echo (TE = 26.8 ms, TR = 2500 ms, matrix size = 256×256, FOV = 2.5×2.5 cm^2^, slice thickness = 0.75 mm, interslice gap = 0.25 mm, 8 echoes, 2 averages) sequence. The eight slices were chosen to cover as much of the brain as possible without compromising the signal to noise ratio of the images. Slices were reproducibly positioned with exactly 1 mm between the notch posterior to the olfactory bulbs and the edge of the first slice (Figure 1A). A set of T_1_-weighted images (TE = 13.0 ms, TR = 600 ms, matrix size = 256×256, FOV = 2.5×2.5 cm^2^, slice thickness = 0.75 mm, interslice gap = 0.25 mm, slice positions identical to those of the T_2_-weighted images, 4 averages) was then acquired over 10 min. A bolus of 0.4 mmol/kg Gd-DTPA (dimeglumine salt, Berlex Canada, 0.5 mmol/mL, 550D) was injected through a 30 gauge tail vein cannula while the mouse remained undisturbed in the magnet. An identical set of contrast-enhanced T_1_-weighted images was obtained immediately following the Gd-DTPA administration, using the same acquisition parameters. Linear phase encoding was used, resulting in the maximum signal being detected 5 min after starting the acquisition.

The animals were examined with MRI pre-PTx/HBSS injection and at days 1, 3, and where possible at day 5 post-injection. Approximately half the transgenic and wild-type mice did not have an imaging session at day 5 post-PTx/HBSS administration due to technical problems with the MR system (Table 1).

**Table 1:** The number of transgenic and wild-type mice that underwent MR imaging at each time point and final histological analysis

**Group**		**Pre-injection**		**Post-injection**			
		**Initial total**	**MRI**	**Day 1**	**Day 3**	**Day 5**	
				**MRI**	**MRI**	**MRI**	**Histology**
**HBSS**	tg	6	6	6	6	4	6
	wt	5	5	5	5	3	5
**Low PTx**	tg	9	9	9	8	4	8
	wt	6	6	6	6	3	6
**High PTx**	tg	7	5	7	6	5	6
	wt	5	5	5	5	2	5

tg: transgenic; wt: wild-type; HBSS: treated with Hanks' Balanced Salt Solution; Low PTx: treated with pertussis toxin at 10 μg/kg; High PTx: treated with pertussis toxin at 20 μg/kg.

### MR image analysis

**Figure 2:**
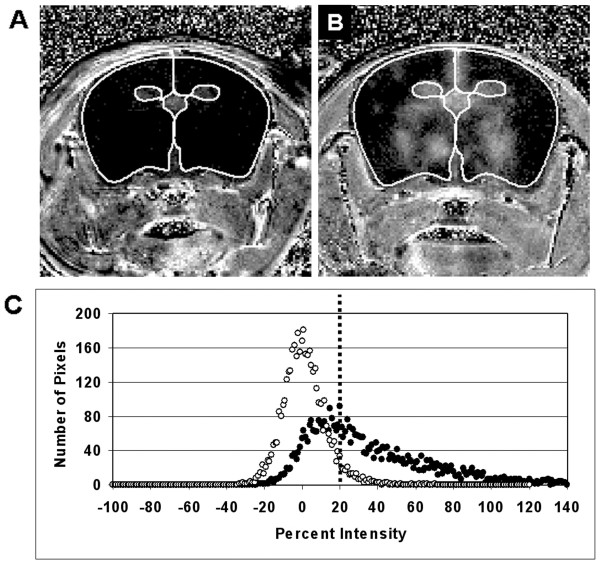
(**A**) Calculated percent enhancement map (fourth slice from the anterior end of the brain; 12mm×12mm) taken from a transgenic mouse pre-PTx injection and (B) a transgenic mouse 5 days post 20 μg/kg PTx injection selecting only the pixels with 20 % or more enhancement. (**C**) Corresponding histograms of the percent intensity within the ROI, where open circles are pre-PTx and filled circles are post-PTx. The threshold used to select enhancing pixels is indicated.

The contrast enhancement on T_1_-weighted images was quantified by calculating percent difference maps as ((post-contrast image – pre-contrast image)/pre-contrast image)×100%. T_2_-weighted images (TE = 80.4 ms) (Figure 1B) were used to define the region of interest (ROI) outlining the brain and omitting the enhancing ventricles (Figure 1C) using Marevisi (National Research Council, Canada) software. These ROIs were superimposed onto the calculated maps of percent enhancement (Figure 2), and histograms of the number of pixels within the ROI versus intensity were generated.

### Tissue processing

After the last imaging session on day 5 post-PTx/HBSS injection, a solution of 70kD lysine-fixable dextran conjugated to fluorescent Texas Red (25 mg/mL in phosphate buffered saline, (0.25 g/kg, 3 moles dye/mol dextran; Molecular Probes, Eugene, OR) was injected into the left ventricle of the heart of each mouse. After one minute the mouse was perfusion-fixed using phosphate-buffered 10 % formalin. The imaged region of the brain was sliced into four sections 2 mm thick. Brain sections were embedded in paraffin and tissue sections 6 μm thick were cut onto glass slides. Laminin was detected by incubating sections overnight using anti-laminin (1:50; rabbit polyclonal antibody Ab-1; NeoMarkers, Fremont, CA) followed by biotin-conjugated secondary antibody, streptavidin-peroxidase, and diaminobenzidine. Sections were incubated with anti-glial fibrillary acidic protein (GFAP; 1:250; rabbit polyclonal antibody; DakoCytomation, Mississauga, ON) to detect astrocytes followed by counter staining for 30 seconds using 4',6-diamidino-2-phenyl indole (DAPI; 1:1000; Sigma, St. Louis, MO) to visualize nuclei. Activated microglia and macrophages were labeled using Bandeira simplicifolia 1 lectin (1:50; Sigma-Aldrich, Saint Louis, MO) and visualized using diaminobenzidine. Adjacent sections were stained with H&E to visualize general histological features. Additional 12 μm thick frozen sections were stained for tight junction protein claudin-5 using rabbit anti-claudin-5 (1:100; Invitrogen, Camarillo, CA, USA), Alexa Fluor donkey anti-rabbit IgG secondary antibody (1:300; Invitrogen), and leukocyte marker CD45 using rat anti-mouse CD45-PE conjugated antibody (1:300; BioLegend, San Diego, CA, USA) followed by counter staining with DAPI.

### Statistics

Data are reported as mean ± standard error of the mean. ANOVA was carried out for calculated difference maps of each slice at day 3 post-PTx/HBSS injection followed by post hoc t tests assuming unequal variance, uncorrected for multiple comparisons. Calculated difference maps of the posterior 6 slices at each time point and percent change in weight between transgenic and wild-type mice before and after PTx/HBSS administration were compared statistically using ANOVA followed by Fisher’s PLSD post hoc test.

## Results

One transgenic mouse that received an initial dose of 20 μg/kg PTx died under anesthetic during imaging on day 3 and a second transgenic mouse that received an initial dose of 10 μg/kg PTx died on day 5.

### MR imaging

**Figure 3:**
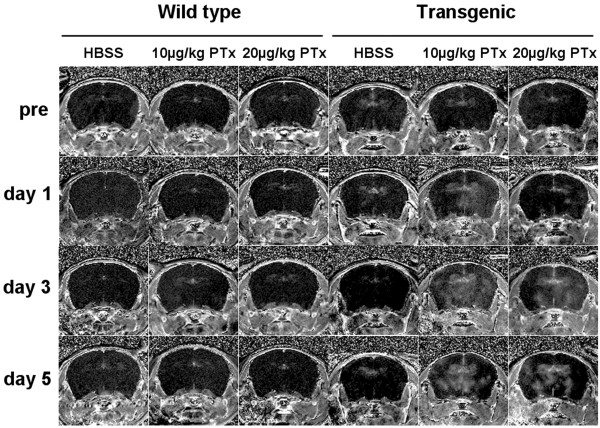
Representative percent enhancement maps calculated from coronal MR images (12mm×12mm) of the fourth anterior slice taken from wild-type and transgenic mice that were administered HBSS, 10 μg/kg PTx, or 20 μg/kg PTx. MR images were obtained pre-injection, and at days 1, 3 and 5 post-injection from the same animal.

**Figure 4:**
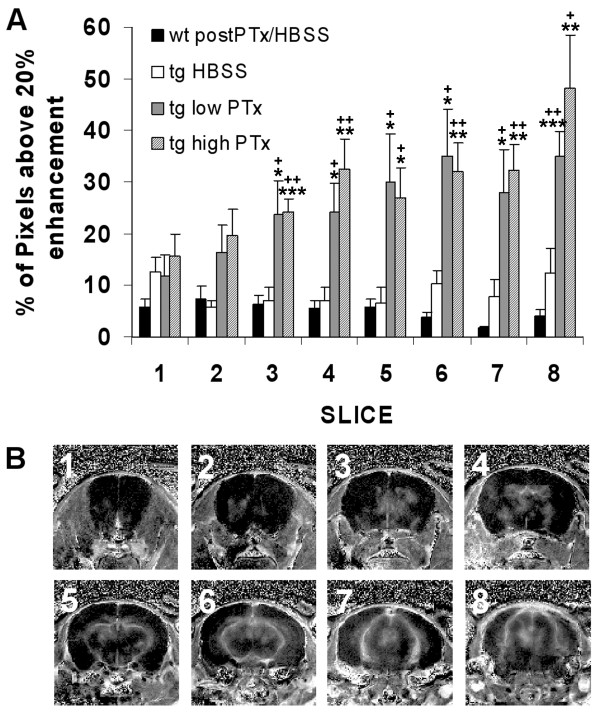
(**A**) The average percentage of enhancing pixels within each brain slice that had intensity enhancement 20 % or greater at day 3 post injection for transgenic and wild-type mice. (**B**) A representative set of calculated difference maps obtained from a transgenic mouse following 20 µg/kg PTx on day 3. Significant differences were found in the 6 most posterior slices for both transgenic (tg) low and high PTx compared to both wild-type (wt) and tg HBSS. * differs from wt, *p* < 0.05, ** *p* < 0.01, *** *p* < 0.001 and + differs from tg HBSS, *p* < 0.05, ++ *p* < 0.01, +++ *p* < 0.001.

Focal areas of enhancement on contrast-enhanced MR images were scattered throughout coronal brain slices of CCL2 transgenic mice that had been injected with PTx (Figures 3, 4). Enhancement was observed predominantly in basal areas of the brain and surrounding the ventricles with fewer regions of enhancement in the cortex. The number and area of focal enhancements increased in more posterior brain slices with greatest enhancement in the midbrain and very little enhancement in the frontal lobes (Figure 4). ANOVA was significant (*p* < 0.001) for slices 3 to 8 and post hoc comparisons were significant in these slices for both transgenic mice that received low and high dose PTx compared to both wild-type mice and transgenic mice that received HBSS. No focal enhancement was observed on contrast-enhanced T_1_-weighted images within the brains of CCL2 transgenic mice that received HBSS or wild-type mice that received either dose of PTx or HBSS (Figure 3).

**Figure 5:**
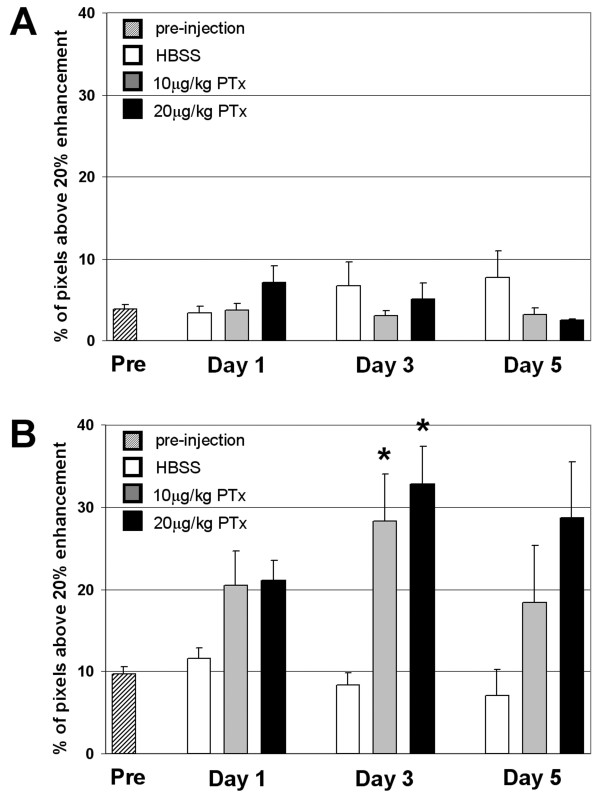
The percentage of pixels in the posterior 6 slices of the brain showing a 20 % or greater increase in intensity on calculated percent enhancement maps for (A) wild-type and (B) transgenic mice pre-injection and days 1, 3 and 5 post HBSS, post 10 µg/kg PTx, and post 20 µg/kg PTx. * differs from HBSS, ***p*** < 0.01.

The histograms obtained from the maps of percent enhancement for wild-type mice and transgenic mice receiving HBSS or before PTx injection showed a normal distribution of intensities, with the number of pixels centered on 2–4 % intensity and typically ranging between ± 20 % intensity (Figure 2). For transgenic mice following PTx administration, the histograms showed a distribution that was skewed to higher intensity (Figure 2) and had a range of intensities extending past 20 %. A relative measure of BBB disruption was determined by calculating the percentage of pixels with a contrast enhancement above 20 % within the brain on calculated percent difference images. Only the posterior six slices were used in these calculations because the slices located more anterior had little contrast enhancement. PTx administration at either dose in CCL2 transgenic mice caused an increase in contrast enhancement that was significant at day 3 (Figure 5). Three days after the administration of PTx at either 10 or 20 μg/kg, there was a significant increase in the percentage of pixels within the brain showing enhancement above 20 % (*p* < 0.01 and *p* < 0.005 respectively, vs mice given HBSS, Figure 5). No significant differences were found between CCL2 transgenic mice pre-PTx/HBSS administration and following HBSS administration at days 1, 3, or 5 or between wild-type mice pre- and post-PTx/HBSS. Contrast-enhanced MR images of transgenic mice pre-PTx/HBSS had a greater percentage of pixels with enhancement greater than 20 % (*p* < 0.0001) than was observed for wild-type mice.

### Clinical signs

There were no significant differences in the change in weight between any of the groups of mice day 1 post-PTx/HBSS injection. Subsequently, a loss in weight was observed only in CCL2 transgenic mice after PTx injection, with greater losses in mice receiving the higher dose. At days 2, 3, 4, and 5 post-PTx/HBSS injection there was a significantly greater loss in weight in CCL2 transgenic mice injected with PTx at low (*p* < 0.05) and high (*p* < 0.005) dose compared to wild-type mice and transgenic mice injected with HBSS. Changes in weight were not significantly different between CCL2 transgenic mice injected with HBSS and wild-type mice at any time point. A significant dose-dependent difference in weight loss was present between transgenic mice that received the high and low doses of PTx at days 3 (*p* < 0.05) and 4 (*p* < 0.05). The largest weight loss was at day 5 in transgenic mice that were administered the low dose PTx (average 5.5 %) and the high dose PTx (average 9.6 %). On average, the trend was that transgenic mice receiving the high dose PTx lost twice as much weight as transgenic mice receiving the low dose PTx. Inactivity was additionally observed in transgenic mice post-PTx injection and death in the case of two transgenic mice.

### Histology

**Figure 6:**
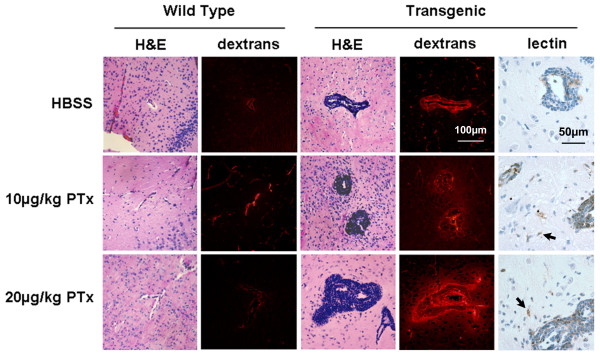
Histological sections stained with H&E to show general features of inflammation and corresponding unstained sections to visualize regions of fluorescence due to dextrans surrounding blood vessels. Sections were taken from representative wild-type and transgenic mice following HBSS or PTx administration and correspond to the final imaging session on day 5 post injection. Original magnification 100×. Additional lectin stained sections identify activated microglia and macrophages from corresponding transgenic mice at 400× magnification. Arrows indicate lectin-labeled cells within the brain parenchyma.

Mice were perfusion-fixed following the last imaging session and tissue sections of the brains were examined for inflammatory cell infiltrates on H&E and lectin stained sections. Sites of BBB opening were revealed by the presence of fluorescent dextrans, which were observed by epifluorescence microscopy. Large numbers of inflammatory cells and fluorescent dextrans were observed within the perivascular spaces of vessels in the brains of all CCL2 transgenic mice (Figure 6). These inflammatory cells surrounded vessels in the cerebral cortex, striatum and white matter, as well as large vessels in the medulla and cerebellar white matter. Inflammatory cells were prominent in the subarachnoid space surrounding large vessels and followed along their superficial sites of penetration. However, not all the large vessels were surrounded by inflammatory cells, rather the inflamed vessels were randomly distributed. Approximately 25 % of the perivascular inflammatory cells were labeled with BS-1 lectin (Figure 6) and no further lectin-positive cells were found beyond the perivascular cluster of cells. Tissue sections from CCL2 transgenic mice also revealed strong claudin-5 staining of the endothelium of vessels with perivascular accumulation of inflammatory cells.

Following PTx administration, inflammatory cells were observed within the brain parenchyma surrounding veins in CCL2 transgenic mice. Fluorescent dextrans were also present in the parenchyma (Figure 6). Rare cells within the parenchyma beyond the cluster of perivascular inflammatory cells were labeled with BS-1 lectin. In transgenic mice that received HBSS, inflammatory cells remained confined to the perivascular space with no evidence of dextrans in brain parenchyma (Figure 6). Based on nuclear morphology approximately 5 % of the infiltrated cells were neutrophils. No inflammatory cells were observed within the perivascular space of brain sections of wild-type mice following PTx or HBSS (Figure 6). Additionally, no BS-1 lectin-labeled cells were present within the brain tissue with the exception of surface ependymal cells.

**Figure 7:**
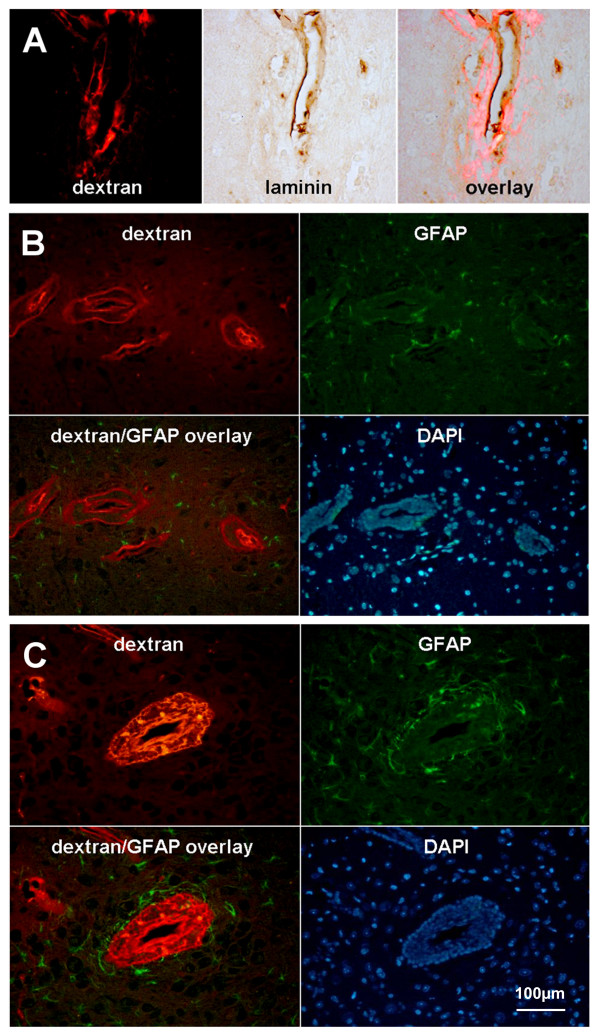
(**A**) Brain section from a CCL2 transgenic mouse that was administered 10 μg/kg PTx and immunolabeled for laminin (brown) shows that the dextrans (red fluorescence) have crossed the basal lamina of the endothelial layer. (**B**) Fluorescence microscopic imaging of a vessel that is leaking dextrans (red) with corresponding staining for GFAP (green) and DAPI (blue) from a CCL2 transgenic mouse that was administered 20 μg/kg PTx. Overlay shows that the dextrans have moved past the astrocytic barrier. (**C**) Comparable images of a non-leaking vessel shows dextrans confined to the perivascular space. Original magnification 100×.

**Figure 8:**
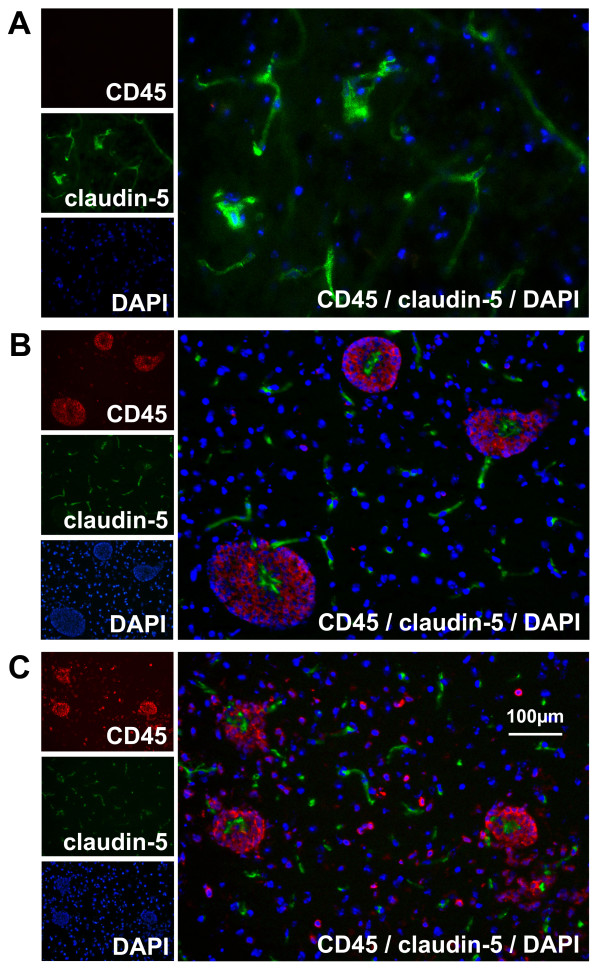
Representative tissue sections stained for tight junction protein claudin-5 (green), leukocyte marker CD45 (red), and DAPI (blue) from (A) a wild-type control mouse, (B) a CCL2 transgenic control mouse, and (C) a CCL2 transgenic mouse post-high dose PTx.

Histologically identifiable perivascular inflammation corresponded well to sites of Gd-DTPA enhancement on the MR images. However, the distribution of these cells and the spread of dextrans were much more confined than the areas of MR enhancement. Staining for nuclei with DAPI confirmed the presence of large numbers of inflammatory cells within the perivascular space. The endothelial barrier was visualized by staining for laminin and dextrans were present across the endothelial barrier in all groups of transgenic mice (Figure 7A). The overlay of GFAP immunolabeling with fluorescent dextrans around inflamed vessels of the brains from CCL2 transgenic control mice showed that dextrans were confined to the perivascular space, and they did not cross the astrocytic barrier (Figure 7C). However, in transgenic mice that received PTx, dextrans moved beyond the astrocytic barrier (Figure 7B). The injection of PTx had no effect on the distribution or intensity in the staining for the tight junction protein claudin-5 in CCL2 transgenic mice (Figure 8).

## Discussion

The breakdown of the BBB and the presence of CCL2 both have a role in the initiation of disease signs in EAE and MS. Therefore, the effects of upregulated CCL2 before and after PTx injection were studied in this model using T_1_-weighted contrast-enhanced MR imaging. As previously described, the upregulation of CCL2 within the CNS of mice causes inflammatory cells to cross the endothelial cell barrier and accumulate within the perivascular space of vessels in the brain; [17,18] but this did not produce any clinical signs of disease. In the present study, contrast-enhanced MR images did not show any evidence of an increase in BBB permeability to a 550D Gd contrast agent in the absence of treatment with PTx. Similiarly, histological examination did not show the presence of larger 70kD dextrans in the CNS parenchyma outside vessels following intravenous administration.

There was no evidence of endothelial dysfunction in vessels surrounded by a perivascular accumulation of inflammatory cells in CCL2 transgenic mice. Staining for tight junction protein claudin-5 demonstrated no difference between CCL2 transgenic mice pre- or post-PTx injection. These observations do not rule out endothelial dysfunction at a previous point in time, as well as the possibility that extravasation occurs in a manner that does not involve disruption of tight junctions. In a previous study of mice with EAE using electron microscopy, there was no evidence of loss of integrity of the BBB during leukocyte migration [21]. This provides supporting evidence for a transcellular migration pathway across the endothelium, with tight junctions remaining intact. Considering a paracellular migration pathway, CCL2 has been found to have a role in the alteration of tight junctions in the endothelium both *in vitro* and *in vivo*[22-24]. A decrease in expression of the tight junction proteins occludin, claudin-5, ZO-1, and ZO-2 was observed following intracerebral injection of CCL2 in mice [23]. The effect of CCL2 on a BBB co-culture model with astrocytes was increased barrier permeability when endothelial cells were CCR2^+/+^ and astrocytes were CCR2^−/−^, but not when endothelial cells were CCR2^−/−^ and astrocytes were CCR2^+/+^[23]. These results suggest that CCL2 acts specifically on the endothelium to cause increased BBB permeability.

The transgenic mouse model used in this study is not a perfect physiologic system as it overexpresses CCL2 resulting in a 100,000 fold increase in the mRNA level of CCL2 within the brain (unpublished data by Toft-Hansen *et al*). Despite this limitation, the CCL2 transgenic mouse model represents a good experimental system in which leukocytes accumulate in the perivascular space, and parenchymal infiltration can be stimulated using PTx. In the present study, no signs of BBB disruption could be visualized on calculated contrast-enhanced MR maps even though inflammatory cells were found to have crossed the endothelium and accumulated in the perivascular space. The overexpression of CCL2 alone was therefore insufficient to produce BBB breakdown, defined in this study as a disruption of the endothelium and glia limitans. The accumulation of fluorescent dextrans in the perivascular space in CCL2 transgenic mice before the administration of PTx or HBSS confirms that the endothelial barrier is permeable to the tracer; however, dextrans were not found to cross the astrocytic barrier. The more confined spread of dextrans in comparison to the areas of MR enhancement suggests either greater sensitivity using MR imaging or greater diffusion of the smaller Gd-DTPA molecule. These observations from histology and contrast-enhanced MR imaging indicate that in the CCL2 transgenic mice there is increased permeability through the endothelial barrier while the astrocytic barrier remains intact and prevents the movement of inflammatory cells, 70kD dextran tracer, and 550D Gd-DTPA contrast agent into the brain parenchyma.

Wild-type mice did not show any signs of BBB disruption following PTx administration. Other studies have shown that PTx causes increased permeability to horseradish peroxidase tracer in a monolayer of brain capillary endothelial cells [25]. Enhanced leakage across the microvasculature in mice due to PTx was also observed following histamine administration [26,27] as well as during the onset of EAE [26]. However, in a previous study of a SJL/J mouse model of EAE, control mice treated with PTx did not show any indications of BBB permeability [28].

When CCL2 transgenic mice were treated with PTx, inflammatory cells infiltrated the brain tissue surrounding blood vessels, dextran tracer leaked into the brain parenchyma, and activated microglia/macrophages were found present in brain parenchyma. The maximum area of focal enhancements on contrast-enhanced MR images was found at day 3 following PTx treatment in CCL2 transgenic mice, suggesting a transient BBB opening in this model, and the decreased contrast enhancement at day 5 indicates that the BBB disruption was beginning to resolve by this time.

Metalloproteinases degrade the extracellular matrix components of the BBB, and metalloproteinase genes were previously found to be significantly upregulated in this CCL2 transgenic mouse model following PTx administration [18]. This occurs along with an increase in proinflammatory cytokines IL-1β and TNF-α, which play a role in leukocyte migration into the CNS [18]. CCL2 transgenic mice showed increased levels of MMP-10, MMP-12, IL-1β and TNF-α even before PTx injection, but the results of the present study show that this is not sufficient to cause BBB permeability. The addition of PTx to the model is the additional stimulus needed to cause BBB breakdown and stimulate the cells accumulated in the perivascular space to cross the astrocytic cell barrier and enter brain parenchyma.

The CCL2 transgenic mice in our study exhibited severe disruption in the BBB that allowed a 70kD dextran tracer to move into the brain parenchyma. Permeability to a large tracer indicates that other large molecules from the blood can escape into the CNS during the inflammatory process. Large serum proteins, such as thrombin, may in turn cause local damage to brain tissue and exacerbate extracellular water accumulation [29-31]. Permeability of the BBB to large molecules in this transgenic mouse model of brain inflammation has important implications for understanding the consequences of and developing treatments for encephalomyelitis.

## Conclusions

The combination of genetic and environmental stimuli resulted in a disruption of the integrity of the BBB in this model of neuroinflammation. The addition of PTx to transgenic mice that overexpresses CCL2 in the CNS resulted in leukocyte migration into brain parenchyma along with increased permeability of tracer and MR contrast agent across the endothelium and glia limitans.

## Abbreviations

BBB, blood–brain barrier; CNS, central nervous system; DAPI, 4',6-diamidino-2-phenyl indole; EAE, experimental autoimmune encephalomyelitis; Gd-DTPA, gadolinium diethylenetriaminepentaacetate; GFAP, glial fibrillary acidic protein; HBSS, Hanks’ balanced salt solution; MRI, magnetic resonance imaging; MS, multiple sclerosis; PTx, pertussis toxin; ROI, region of interest.

## Competing interests

The authors declare that they have no competing interests.

## Authors’ contributions

AES was involved in data acquisition, data analysis, statistical analysis, study design and drafted the manuscript. RB participated in the acquisition of MR images and statistical analysis. MRD contributed to the histological analysis and critically reviewed the manuscript. HTH participated in study design and some of the experiments involving PTx injection to transgenic mice. TO participated in study design and bred the transgenic mice used in the study. RK contributed with immunofluorescent staining of claudin-5. JP participated in study design, data analysis and helped to draft the manuscript. All authors have read and approved the final version of the manuscript.
